# Infant Feeding

**Published:** 1890-08

**Authors:** W. I. Thayer


### INFANT FEEDING.

W. I. Thayer, M. D.

An exceedingly entertaining editorial in the June Homœopathic Physician states that "the artificial feeding of infants is one of the most difficult problems with which the physician has to deal," and "if one could rely upon the various commercial foods with which the market is flooded, the question would at once be solved."

This subject of infant feeding is indeed a difficult problem—has been—to solve, if one is to rely upon most of the baby-foods in the market, or, expects to obtain the best results in giving to a baby cow's milk, no matter how much reduced with water or mixed with crushed barley.

Dr. Clark refers to the *casein*, "the curd-forming matter," and it is just here that cow's milk, pure and simple, is *not* as good a food for an infant as though the "*curd*" had been put into such a condition as to be *easily digested.*

The *only* nitrogenous constituent of human or cow's milk is that protein compound known as *casein!* If a child cannot digest and appropriate this nitrogenous substance in either milk it is *not* going to get *full nutrition*, and will slowly starve!

Why is it that human milk will digest so much easier than will cow's milk ?

Chiefly, and almost wholly, on account of the different physical characteristics of the two caseins !

When the casein of *human* milk comes into contact with the chlorohydric acid of the gastric juice it is turned into a *light, flaky* coagulum, easily attacked by the peptic ferment and farther comminuted.

On the contrary, the casein of cow's milk, when presented to the gastric juice, coagulates into rounded masses, which *prevents* the peptic ferment from seizing hold of as many surfaces as are presented in the protein compound of mother's milk, and hence slower disintegration of the curd must follow, and less of it is peptonized or gastricly digested, and quite a portion of this *tough, leathery* curd must traverse the intestinal canal, rasping its way to the anus, and setting up various bowel lesions. These are physiological facts that can be demonstrated in a chemical laboratory—as well as in the digestive tract—and are not fanciful theories.

The chemico-physical properties between the two curds are very different. What will dissolve one will but feebly effect the other.

Contrast the digestive apparatus of an infant with but *one* stomach to dispose of its food and the same machinery in a calf with *four* stomachs, and which can get up and run as soon as it sees daylight, and tell me, did Providence compose the same natural pabulum for these two animals when He has made their physicial conditions so very, very different ?

Nay, verily !

The average amount of casein found in forty-three women was 1.046 per cent. of the whole.

Thirteen different observers find the average per cent. of casein in cow's milk to be 3.022.

A marvelous difference, and if it would digest as easily as the same protein substance of mother's milk, *there is too much of it,* for mother's milk contains of nitrogenous matter about 17 per cent., while cow's milk will average 25.59 per cent.—some claim from 28 to 30 per cent.—so that in cow's milk we get, first, too much casein by a large per cent., and what we have after reducing with water, is *not* readily digestible, as its refractory properties have *not* been changed in the least.

It is pertinent to inquire, Can cow's milk be so manipulated as to be a good substitute for mother's milk by balancing its protein substances and so operating upon its tough, cheesy casein as to make the whole mass like mother's milk and easy of digestion ?

The answer must be in the affirmative, because it is done in great quantities. Certain it is that it is not within the purview of the physician to do this, and the nurse could not come out twice alike in any pre-digesting of cow's milk, and, as has been shown, it is necessary to *tear* the cow-casein somewhat to pieces to make it of easy digestion.

While Dr. Clark's statements hold true that the large mass of baby-foods "are generally not to be trusted," I hold that there has recently been offered to the confidence of the profession one that meets all of the suggestions of this paper and is a good substitute for mother's milk, as I have clinically proven in several cases.

Two cases of inanition resulting from a *poor quality* of mother's milk which failed to nourish the infants.

Another case, in a twin boy, who was fed upon the very best quality of cow's milk, but he could not digest it, for reasons stated above. Also cases of cholera infantum ; all improved *immediately* upon feeding them upon an artificial food recently placed upon the market and known by the peculiar name of Lacto-Preparata, composed of partly predigested and desiccated cow's milk, whose protein substances had been made to *balance* like human milk, by adding sugar of milk, as suggested by Dr. Clark.

One of the infants weighed at six and a half months sixteen pounds. The second gained four pounds in two months. The twin boy could digest this artificial food just as easily as did his twin sister its mother's milk.

One may boil flour for ten days and he will never convert it into dextrine or soluble starch. Such a conversion *requires* a temperature of 350° F. for eight hours, and as water boils at 212° F. he is short in changing power by 138 degrees.

Again. Malt sugar, being soluble in water, it will not to any appreciable extent prevent the coagulation of the casein of cow's milk, and in the addition of any cereal flour that has not been converted into dextrine, quite as indigestible a substance has been added to the milk as the casein itself.

I hold that an artifical food made upon the above principles will digest as easily as mother's milk and *nutrify every tissue*, which is not always the case, in *poorly-fed* mother's milk ; and one will find in the above food all that is required in a good and reliable artificial food for infants under eight months of age.

[We sincerely trust that Dr. Thayer is correct regarding the food of which he speaks. We fear, however, that he will find it of benefit in some cases, and of no value in others. Predigested foods have not as yet met the demands of the system; in many cases they have proved of no value.—G. H. C.]

